# Interdisciplinary Physician-Pharmacist Medication Review for Outpatients With Heart Failure: A Subanalysis of the PHARM-CHF Randomized Controlled Trial

**DOI:** 10.3389/fphar.2021.712490

**Published:** 2021-09-07

**Authors:** Pia M. Schumacher, Nina Griese-Mammen, Juliana Schneider, Ulrich Laufs, Martin Schulz

**Affiliations:** ^1^ Department of Medicine, ABDA–Federal Union of German Associations of Pharmacists, Berlin, Germany; ^2^ Department of Cardiology, Leipzig University Hospital, Leipzig, Germany; ^3^ Drug Commission of German Pharmacists, Berlin, Germany; ^4^ Institute of Pharmacy, Freie Universität Berlin, Berlin, Germany

**Keywords:** heart failure, medication discrepancy, medication reconciliation, interdisciplinary care, community pharmacy service, medication plan

## Abstract

**Aims:** Patients with chronic heart failure (CHF) require polypharmacy and are at increased risk for drug-related problems. Interdisciplinary physician-pharmacist medication review may improve drug treatment. Our goal was to analyze the changes from the physician-documented medication plan (MP) and patient-stated medication to an interdisciplinary consolidated MP (CMP).

**Methods:** This pre-specified subanalysis of the PHARM-CHF randomized controlled trial analyzed the medication review of CHF patients in the pharmacy care group. Community pharmacists compared the MP with the drug regimen stated by the patient and consulted with physicians on identified discrepancies and other medication-related problems resulting in a CMP.

**Results:** We analyzed 93 patients (mean 74.0 ± 6.6 years, 37.6% female), taking a median of ten (IQR 8–13) drugs. 80.6% of patients had at least one change from MP to CMP. We identified changes in 32.7% (303/926) of drugs. The most common correction was the addition of a drug not documented in the MP to the CMP (43.2%). We also determined frequent modifications in the dosing regimens (37.6%). The omission of a drug documented in the MP but left out of the CMP accounted for 19.1%. Comparing patient-stated medication to CMP, the current drug regimen of patients was changed in 22.4% of drugs.

**Conclusion:** The medication review resulted in changes of medication between MP and CMP in most of the patients and affected one-third of drugs. Structured physician-pharmacist interdisciplinary care is able to harmonize and optimize the drug treatment of CHF patients.

## Introduction

Chronic heart failure (CHF) affects approximately 1–2% of the population in the developed countries and is highly morbid and costly with a growing impact on public health (Groenewegen et al., 2020). Patients with CHF benefit from several drug classes and the complexity of the pharmacotherapy increases with disease progression. Additionally, numerous comorbidities add to the complexity of the HF syndrome. This situation increases the risk of adverse outcomes due to polymedication, inappropriate prescribing (Goyal et al., 2020), medication errors, medication non-adherence (Schulz et al., 2019), and other drug-related problems (DRP) that can potentially exacerbate HF (Tsuyuki et al., 2001).

To support outpatients in their complex daily therapy regimen, general practitioners (GPs) or specialists provide medication schedules or medication plans (MP). International studies have shown that not all the patient’s current medication is recorded and thus the physician-documented medication is often incomplete (Bedell et al., 2000; Bikowski et al., 2001; Schnipper et al., 2018). In Germany, over 90% of the MP did not comply with the medication actually being taken by the patient (Waltering et al., 2015; Rose et al., 2018). Incomplete MP results from missing documentation in the patient file, incorrect transfer into the MP, insufficient communication between GPs, specialists and pharmacists, as well as undocumented use of non-prescription drugs (Schmiemann et al., 2012). This is a major challenge as the medications stated by the patient, but not documented by the physician, were often associated with a high risk for falls, hospitalization, or drug-drug interactions (Rose et al., 2018). In particular, drugs acting on the cardiovascular system are prone for deviations; therefore, CHF patients are commonly affected by drug discrepancies (Ekedahl et al., 2011; Rose et al., 2018; Giannini et al., 2019; Imfeld-Isenegger et al., 2020). Additionally, the frequency of discrepancies increased with patient age, the involvement of a specialist, and the patient’s unfamiliarity with the medication (Bedell et al., 2000).

Different types of medication discrepancies have been classified: drug omission and drug addition, as well as deviations in strength, frequency, number of units, or daily dosage (Almanasreh et al., 2019). The potential to harm the patient is clinically relevant (Imfeld-Isenegger et al., 2020).

The pharmacist-led medication reconciliation is a strategy for more accurate medication lists, but there are still frequently discrepancies (Stewart und Lynch 2014). The interdisciplinary collaboration of physicians and pharmacists is a successful intervention to reduce these discrepancies (Arnold et al., 2015; Elliott et al., 2019; Holland, 2015). The interdisciplinary consolidation between the dispensing pharmacist and the attending physician based on the DRP identified in a medication review is an essential process to determine an optimal and safe medication scheme (Yates et al., 2020). Technical solutions such as electronic prescribing systems could not thus far eliminate the variations between the different documentations of medications (van Stiphout et al., 2018; Ernst et al., 2001).

Previous trials focused on identifying and defining the discrepancies in medication, comparing physician’s documentation with patient-stated medication or electronic pharmacy records (Bedell et al., 2000; Bikowski et al., 2001; Ekedahl et al., 2011; Waltering et al., 2015; Rose et al., 2018). Data on the agreed changes that result from performing a medication review followed by a consolidation of the MP by the physician and the pharmacist in the outpatient setting is scarce. Thus, we aimed to identify the impact of an interdisciplinary consolidation on the drug regimens of CHF outpatients.

## Materials and Methods

### Study Design

**FIGURE 1:**
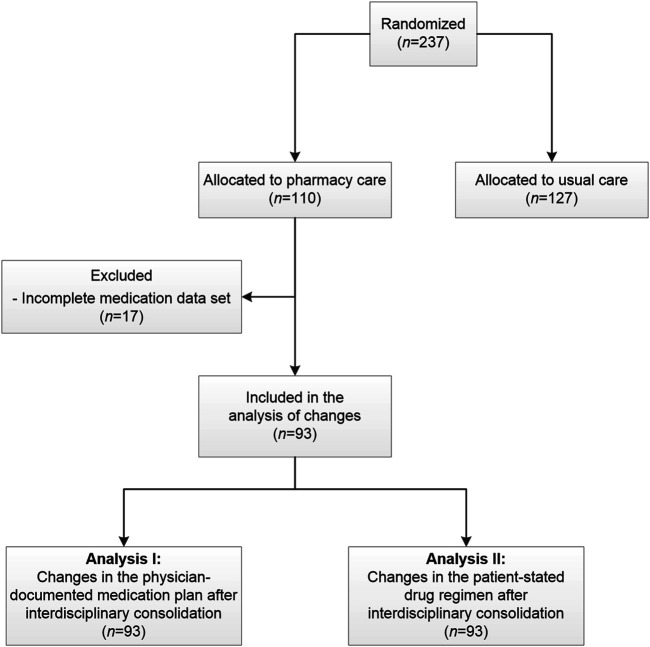
Subanalysis flowchart.

**FIGURE 2:**
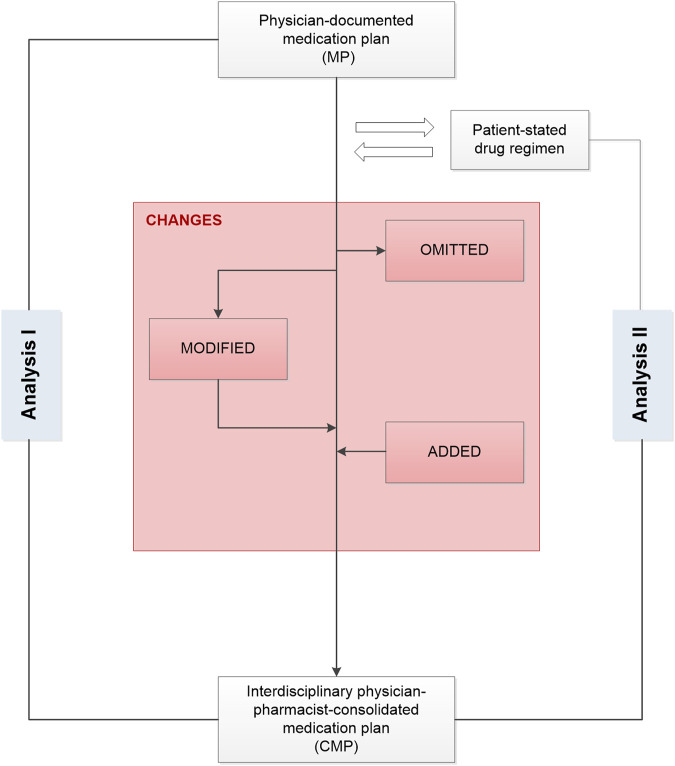
Development of an interdisciplinary consolidated medication plan after comparing the drug information in the medication plan documented by the study physician with the results of the interview between the pharmacist and the patient in a medication review. CMP, consolidated medication plan; MP, medication plan.

The current analysis is based on data collected in the PHARM-CHF randomized controlled trial. The study design and intervention have been described in detail in previous publications (Schulz et al., 2019; Laufs et al., 2018; Schulz et al., 2020). To summarize, PHARM-CHF was an investigator-initiated, prospective multicenter randomized controlled trial in Germany with blinded adjudication of hospitalization events. The recruited patients aged 60 years and older had an established diagnosis of CHF, stable CHF medication including a diuretic, and HF hospitalization within the last 12 months or increased B-type natriuretic peptide or N-terminal pro-B-type natriuretic peptide concentrations. Randomization to the pharmacy care group or usual care group occurred via a secure web interface tool. One hundred and ten patients were assigned to the pharmacy care group and 127 patients to the usual care group (Figure 1) (Schulz et al., 2020). Patients allocated to the pharmacy care group received a comprehensive medication review (type 2a according to the Pharmaceutical Care Network Europe classification (Griese-Mammen et al., 2018)) performed by community pharmacists. This procedure aims at generating a complete and optimal physician-pharmacist consolidated MP (CMP). It is a structured compilation and comparison of the patient’s entire current medication, that is, physician’s documentation, pharmacy records, and a patient interview about the medication used at home, performed by a pharmacist (Laufs et al., 2018). The physicians provided the pharmacists their medication documentation via a secure online tool (electronic Case Report Form [eCRF]). The pharmacists invited the participating patients to an interview and asked them to bring their current medication from home. Additionally, the pharmacists provided the pharmacy records of the patient, if available. During the patient interview, medication recorded in the eCRF was compared with the patient-stated medication/pharmacy records. The patient was also asked about the current dosing regimens and the reasons for missing or additional drugs (Supplementary Material S1). The identified DRP were discussed with the physician if necessary to optimize and harmonize the drug regimen (Figure 2). The pharmacist then entered the CMP into the eCRF and the physician approved the consolidated adjustments digitally. Based on the CMP, the patients received the filled dosing aids at their (bi-)weekly visits to the pharmacy. They also received a printout of the CMP for their own documentation. The patients allocated to the usual care group did not get this intervention and filled the prescriptions in pharmacies of their choice as usual.

PHARM-CHF (ClinicalTrials.gov identifier: NCT01692119) was conducted according to the principles stated in the current version of the Declaration of Helsinki, International Conference on Harmonization Good Clinical Practice, and according to the local and national regulations. The documented approvals from the independent ethics committees were obtained for all participating centers and written informed consent was obtained from all patients (Laufs et al., 2018).

The aim of the PHARM-CHF main study was to investigate whether regular contact with the local pharmacy and (bi-)weekly dosing aids based on a physician-pharmacist CMP improve medication adherence (primary efficacy endpoint) and reduce hospitalization and mortality (primary safety endpoint) in elderly CHF patients compared to usual care (Schulz et al., 2019).

In this subanalysis, we analyzed the agreed changes of medication after interdisciplinary consolidation of the medication of patients receiving pharmacy care. Thus, we aimed at identifying the changes from MP to CMP and whether those changes were based on information stated by the patient (“Analysis I”). In addition, we analyzed the changes from the patient-stated drug regimen to the CMP (“Analysis II”) (Figure 1).

### Analysis I: Changes in the Physician-Documented Medication After Interdisciplinary Consolidation

We compared the physician-documented MP with the CMP to identify agreed changes based on the interdisciplinary consolidation such as omissions and additions as well as modifications of a drug (Figure 2). For instance, a drug identified in the CMP but not in the MP was categorized as “drug addition.” A drug not identified in the CMP but documented in the MP was categorized as “drug omission.” The category “drug modified” summarized the changes in the strength, frequency, number of units, and/or daily dosage. In this case, the drug’s active ingredients were identical on both plans but had one or more modifications between the MP and the CMP. Any difference between the information in the MP and the CMP was defined as an agreed change. In case the CMP was different from the initial MP, we compared the patient’s current drug regimen with the information in the CMP.

### Analysis II: Changes in the Patient-Stated Drug Regimen After Interdisciplinary Consolidation

As a secondary aspect, we analyzed the impact of the interdisciplinary consolidation on the patient-stated medication to identify modifications and optimizations of the current regimen. We compared the entire medication stated by the patient during the medication review with the CMP. The changes were classified similar to the terminology described above: a drug identified in the interview but not in the CMP was counted as “omission,” a drug identified in the CMP but not in the patient interview was counted as “addition,” and “modifications” were changes in the strength, frequency, number of units, and/or daily dosage.

### Statistical Analyses

We used IBM SPSS (version 25) to uncover any predictors of medication changes between the physician-documented MP and CMP. The Shapiro-Wilk test was applied to check the distribution of variables. Association between the age, number of drugs, care level, or NYHA class and medication changes was tested with Spearman’s correlation. We applied the Mann-Whitney *U* test to compare the values of the changes depending on the physician’s profession (GP, cardiologist), sex, or participation in an HF disease management program (HF-DMP). The statistical significance was determined with an alpha value of 0.05.

## Results

For the analysis of changes after interdisciplinary consolidation, complete medication data sets were available for 93/110 (84.5%) patients (74.0 ± 6.6 years, 37.6% female) of the pharmacy care group (Table 1). The patients had an overall median of ten (IQR 8–13) drugs, considering all the sources of information (MP, patient interview, and CMP). Examining the MP, the patient interview, and the CMP, we identified a total of 985 drugs in at least one of these sources.

**TABLE 1:** Baseline characteristics of the analyzed patients (*n* = 93).

Characteristic	
Age, mean ± SD, years	74.0 ± 6.6
Median (IQR)	75.0 (69–79)
≥75 years, n (%)	50 (53.8)
Female sex, n (%)	35 (37.6)
BMI^a^, mean ± SD, kg/m^2^	29.1 ± 5.3
LVEF <40%, n (%)	21 (22.6)
LVEF 40–49%, n (%)	35 (37.6)
LVEF ≥50%, n (%)	37 (39.8)
NYHA classes I/II, n (%)	38 (40.9)
NYHA classes III/IV, n (%)	55 (59.1)
Time since last hospitalization for HF, mean ± SD, years	0.35 ± 0.73
Different co-morbidities, mean ± SD	7.4 ± 2.5
Hypertension, n (%)	90 (96.8)
Hyperlipidemia, n (%)	79 (84.9)
Coronary heart disease, n (%)	66 (71.0)
Diabetes	54 (58.1)
Atrial fibrillation, n (%)	52 (55.9)
Chronic kidney disease, n (%)	40 (43.0)
Medication, n (%)	985 (100)
Per patient, median (IQR)	10 (8–13)
No. of drug packages, mean ± SD	8.7 ± 3.1
No. of single doses/day, mean ± SD	10.5 ± 3.9
No. of drug intakes/day, median (IQR)	3.0 (2–3)
HF medication, n (% of medication)	304 (30.9)
ACEi/ARB, n (% of HF medication)	80 (26.3)
Beta-blockers, n (% of HF medication)	90 (29.6)
MRA, n (% of HF medication)	40 (13.2)
Diuretics^b^, n (% of HF medication)	94 (30.9)

ACEi, angiotensin-converting enzyme inhibitor; ARB, angiotensin receptor blocker; BMI, body mass index; HF, heart failure; IQR, interquartile range; LVEF, left ventricular ejection fraction; MRA, mineralocorticoid receptor antagonists; NYHA, New York Heart Association (functional class); SD, standard deviation.

a The body mass index (BMI) is the weight in kilograms divided by the square of the height in meters.

b All patients received a diuretic.

### Analysis I: Changes in the Physician-Documented Medication After Interdisciplinary Consolidation

**FIGURE 3:**
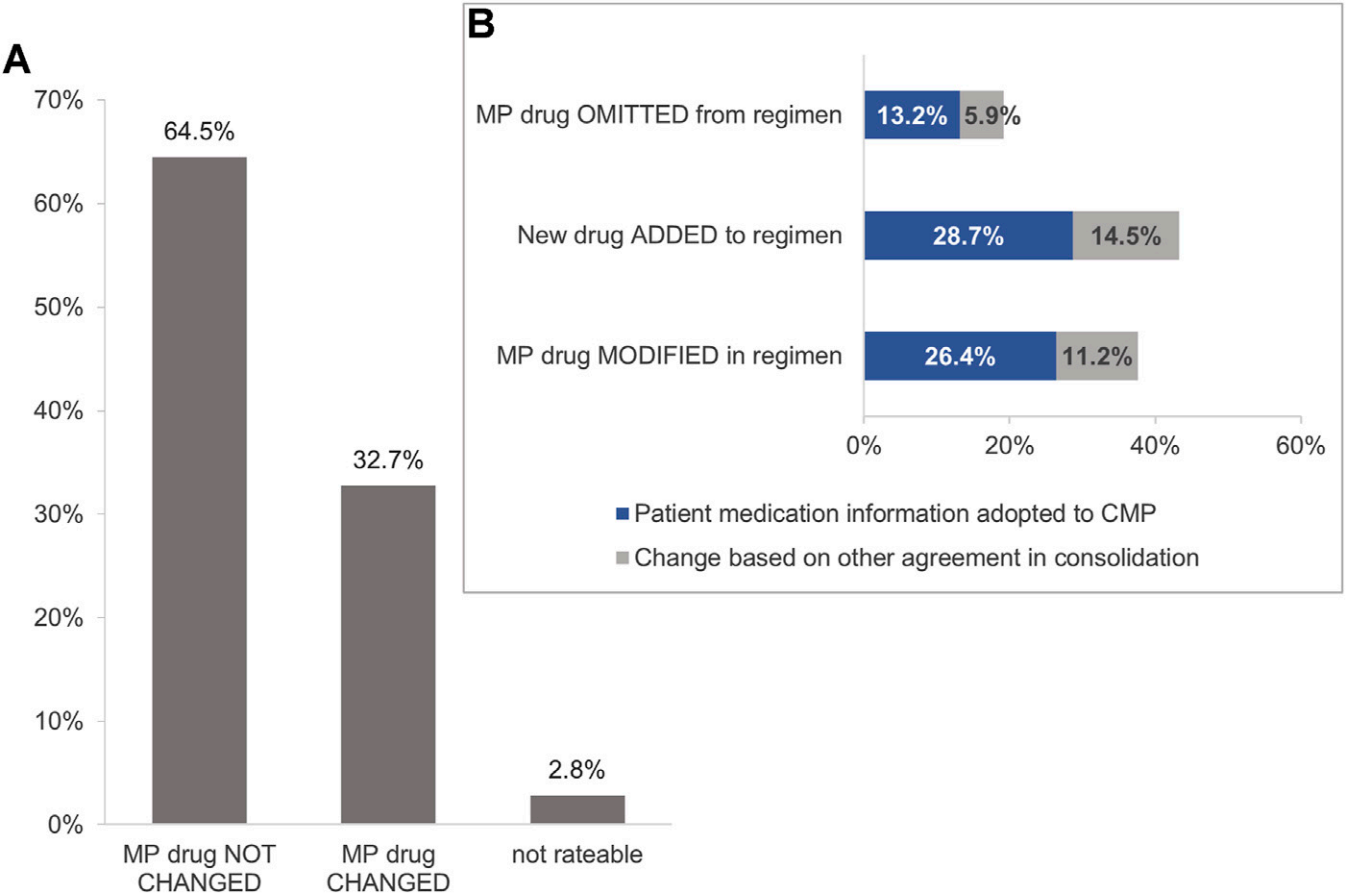
**(A)** Impact of interdisciplinary consolidation on the physician-documented drug regimen of heart failure patients (*n* = 926 drugs*). *Fifty-nine drugs identified only in the patient interview were not included in Analysis I. CMP, consolidated medication plan; MP, medication plan. **(B)** Changes in the drug regimen resulting from the medication review (*n* = 303 drugs). Variations in the percentages or from 100% are due to rounding deviations in values.

Of the 985 drugs identified, 926 (94.0%) were identified in the MP and/or in the CMP. At least one change in the medication between the MP and the CMP was identified in 80.6% (*n* = 75) of the patients. Overall, the number of medication changes ranged from 0 to 20 per patient and we observed a median of 2 (IQR 1–4) without significant sex-specific differences. The impact of the physician-pharmacist consolidation is shown in Figures 3A,B. Additionally, we present an overview on examples of identified changes in Table 2.

**TABLE 2:** Examples of the medication reviews resulting in omissions, additions, or modifications of the medication plan.

Type of change	Results of the interdisciplinary consolidation	Examples
**Omission (*n* = 58, 19.1%)**		
Drug stated in MP not identified in CMP	Drug documented by physician and stated by patient but not adopted into CMP	Discontinuation of potassium capsules while taking spironolactone
	Drug documented by physician but discontinued by patient and also not adopted into CMP	Discontinuation of amiodarone after adverse drug effects (nausea, circulation problems, dizziness, and rashes)
		Discontinuation of temporarily used enoxaparin
**Addition (*n* = 131, 43.2%)**		
Drug not stated in MP but identified in CMP	Drug not documented by physician but adopted into CMP as stated by the patient	Inclusion of buprenorphine, PRN nitroglycerin spray, or insulin into the CMP for completeness
	Drug not documented by physician but identified in CMP: switch of the active ingredient that was stated by the patient	Adoption of atorvastatin instead of simvastatin into the CMP for improved effectiveness
**Modification (*n* = 114, 37.6%)**		
Drug stated in MP and CMP but changed in strength, frequency, number of units, and/or daily dosage	Modification of MP dosing regimen due to underdosing	Increased dose of metoprolol succinate 23.75 mg 1 - 0 - 0 to 1 - 0 - 1 for improved effectiveness
	Modification of MP dosing regimen to avoid tablet splitting (e.g., because of loss of drug substance, stability problems, incorrect dosing, and handling problems)	Change of furosemide 20 mg 0.5 - 0 - 0.5 to 1 - 0 - 0, to avoid tablet splitting and exposure to light and humidity outside original packaging
		Change of metoprolol 100 mg 0.5 - 0 - 0.5 to metoprolol 50 mg 1 - 0 - 0 to avoid tablet splitting
	Modification of MP dosing regimen to facilitate intake	Shifting time of intake, e.g., aspirin 100 mg from noon to morning, to support medication adherence
	Modification of MP dosing regimen to avoid adverse drug events	Change of furosemide 20 mg 1 - 1 - 0 to 40 mg 1 - 0 - 0 to avoid urinary urgency in the evening/at night

MP, physician-documented medication plan; CMP, interdisciplinary consolidated medication plan; PRN, *pro re nata:* as needed.

Of the 926 drugs identified in the MP and/or in the CMP, 32.7% (*n* = 303) were altered between the MP and the CMP (Figure 3A). These changes were an omission of drugs in 19.1% (*n* = 58), meaning a drug stated in the original MP was not adopted in the CMP (Figure 3B). In contrast, 43.2% (*n* = 131) of identified drugs were added to the CMP as they were not documented originally in the physician’s MP (Figure 3B). In another 37.6% (*n* = 114) of drugs, the consolidation resulted in a modification of a drug (Figure 3B; Table 2).

Approximately 68% (*n* = 207) of the changes matched the current drug regimen stated in the patient interview (Figure 3B, blue bars). The omitted drugs were not identified in the medication review (13.2%, *n* = 40) and thus not adopted into the CMP. In 26.4% (*n* = 80), the changes were modifications according to the findings in the medication review, such as the method of intake or the drug strength. The drug additions to the CMP were based on the findings in the medication review in 28.7% (*n* = 87) of the changes (Figure 3B). In 31.7% (*n* = 96), the changes were based on the agreements within the process of consolidation between physicians and pharmacists (Figure 3B, grey bars). Of those changes, 21.9% (*n* = 21) resulted from the relevant DRP that were identified by the pharmacist in the medication review.

**FIGURE 4:**
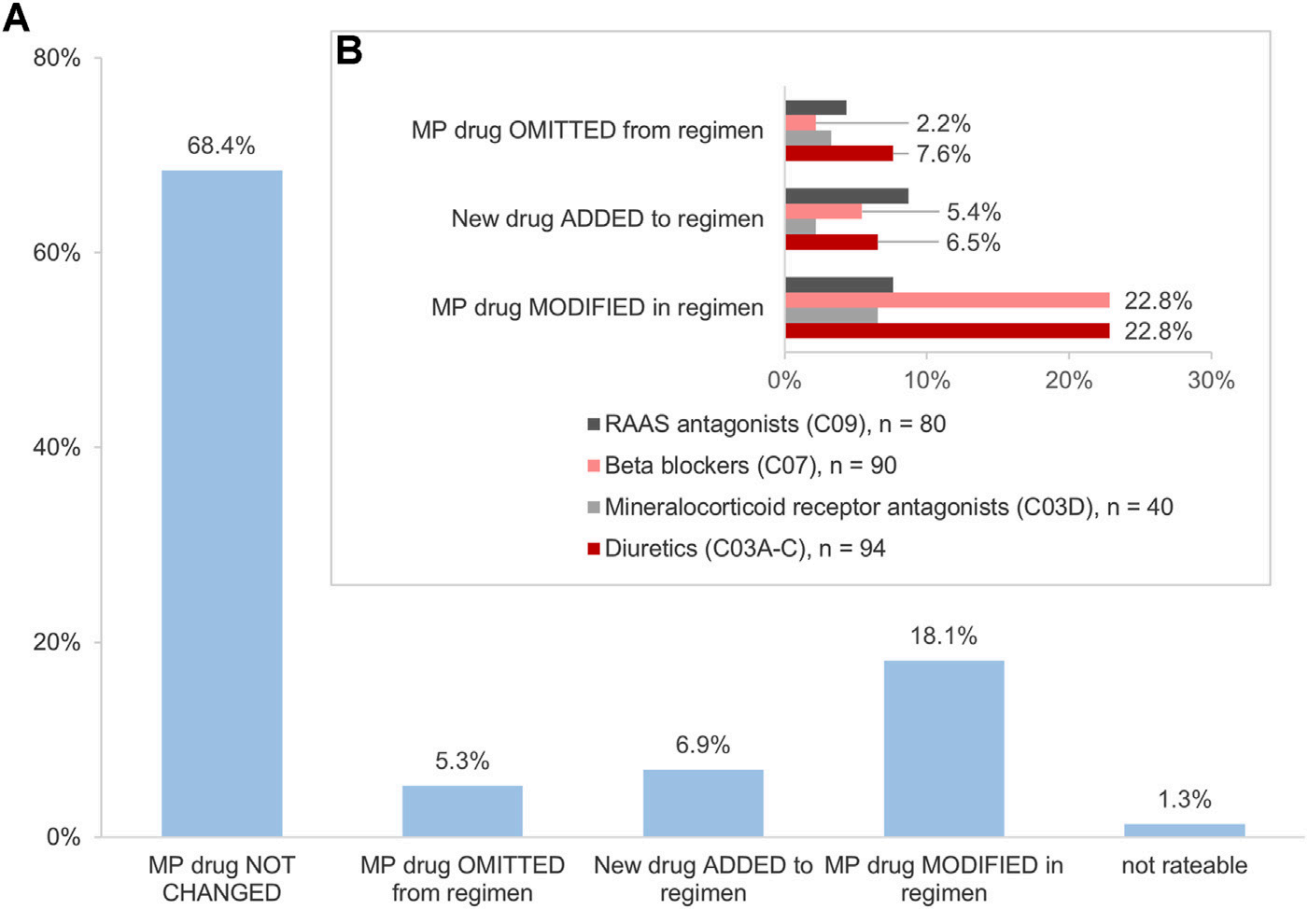
**(A)** Impact of the interdisciplinary consolidation on the heart failure medication (*n* = 304 drugs). **(B)** Changes in the heart failure medication (*n* = 92 drugs). Variations in the percentages are due to rounding deviations in values.

**FIGURE 5:**
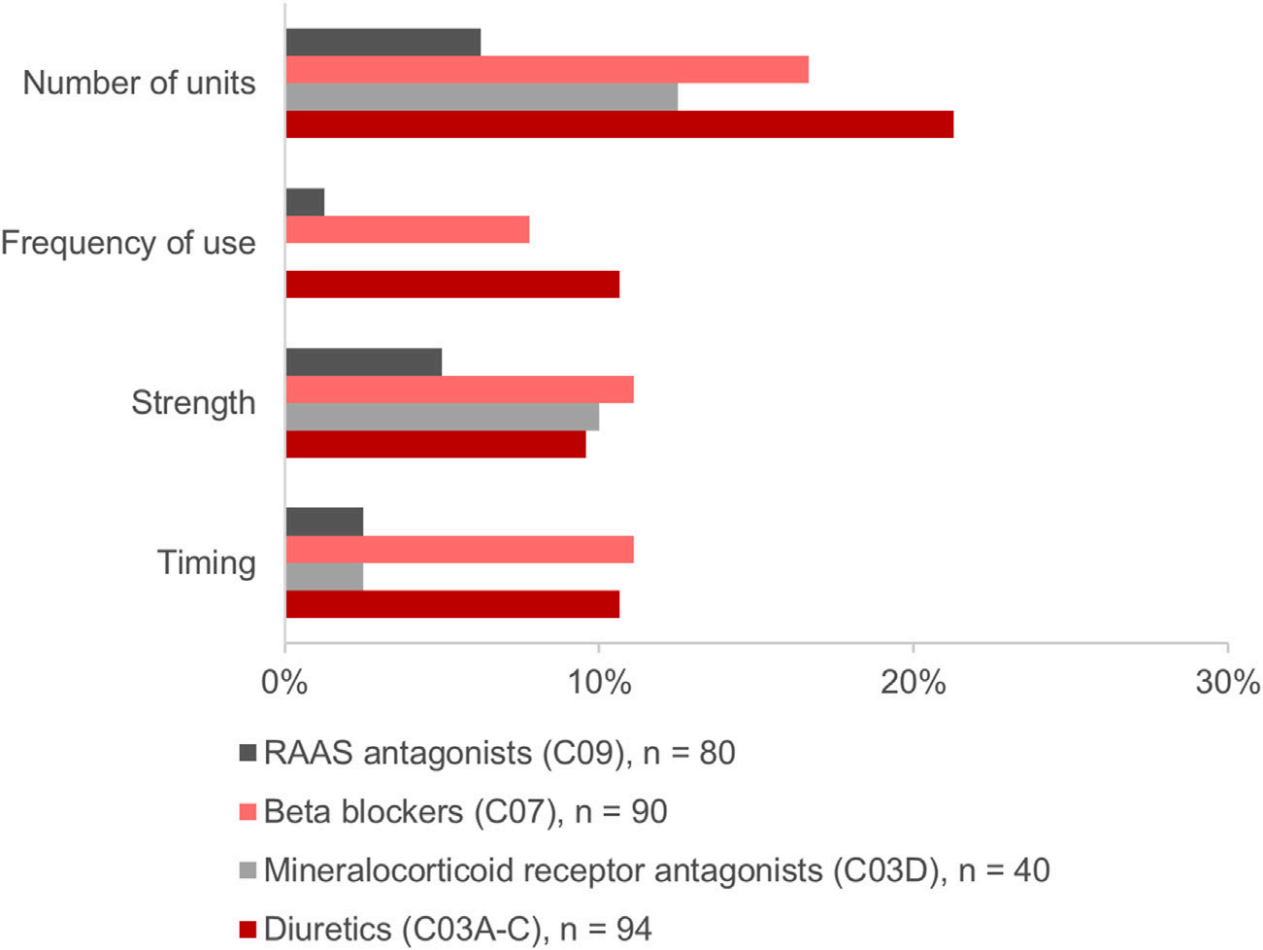
Modifications in the heart failure medication comparing the physician’s medication plan (MP) and the consolidated medication plan (CMP).

Of the above-mentioned drugs identified (*n* = 985), 307 (31.2%) were HF medications. Of those, 304 (99.0%) were identified in the MP and/or in the CMP. This included 26.3% (*n* = 80) agents acting on the renin-angiotensin system, 29.6% (*n* = 90) beta-blockers, 13.2% (*n* = 40) mineralocorticoid receptor antagonists (MRA), and 30.9% (*n* = 94) diuretics. The changes in the HF medication occurred in 30.3% (*n* = 92) of drugs (Figure 4A). Diuretics accounted for 37.0% (*n* = 34) and beta-blockers for 30.4% (*n* = 28) of the changes and were, thus, especially affected by the consolidation process (Figure 4B). Most frequently, the modifications in the number of units such as the increase or decrease of the number of tablets per day were identified for diuretics and beta-blockers, respectively (Figure 5). Additionally, 10.0% (*n* = 8) of agents acting on the renin-angiotensin system were missing in the MP and added to the CMP. The majority of changes in HF medication (71.7%, *n* = 66) concurred with the information from the patient interview which was then adopted into the CMP.

The number of drugs correlated significantly with the number of changes (r_s_ = 0.611; *p* < 0.001) in the CMP. We observed that for the MP documented by the cardiologists (*n* = 36), a significantly higher number of changes were made in the CMP compared to those documented by the GPs (*n* = 57) (median: 4.5 [IQR 1.5–6.0] vs 2.0 [IQR 0.0–4.0], Mann-Whitney test, *U* = 556.0, *p* < 0.001). For the patients taking part in an HF-DMP (*n* = 27), a significantly lower number of changes in the CMP were observed compared to the patients who did not participate in such a DMP (*n* = 66) (median: 1.0 [IQR 0.0–2.5] vs 3.0 [IQR 1.0–5.0], Mann-Whitney test, *U* = 550.0, *p* = 0.004). We found no association between age, NYHA class, or care level and the number of changes.

### Analysis II: Changes in the Patient-Stated Drug Regimen After Interdisciplinary Consolidation

We identified 945 drugs (95.9%) that were stated in the patient interview and/or the CMP. A further 40 drugs (4.1%) were only identified in the MP. We found that 212 (22.4%) drugs changed between the patient-stated medication and the CMP. After the interdisciplinary consolidation, 74.2% (*n* = 69) of patients had at least one change in their current medication therapy (median 3, IQR 1–4). The drugs identified in the patient interview but not adopted into the CMP accounted for 36.3% (*n* = 77) of the changes. The omissions belonged mostly to anti-inflammatory and anti-rheumatic substances, mineral supplements, and diuretics, including MRA.

We identified additions in 22.2% (*n* = 47) of the drugs and modifications in 41.5% (*n* = 88). Medications added to the CMP were mainly drugs for obstructive airway diseases, lipid modifying agents, and diuretics/MRA. The drugs for obstructive airway diseases and for diabetes as well as diuretics/MRA were modified frequently by altering the units and the timing or increasing the dose or the strength. In 26.9% (*n* = 57) of the changes, the physician-pharmacist consolidation resulted in the adoption of the physician-documented therapy regimen into the CMP instead of the patient’s current regimen. The reasons for changing the current patient regimen were, for example, improving patient safety (Table 2).

## Discussion

We assessed patient-physician-pharmacist collaborations with regard to a consolidated medication plan (CMP). This approach considered the physician-documented medication plans (MP) and the patient-stated medication taken at home in a cohort of CHF outpatients. To the best of our knowledge, this is the first analysis of the extent of the agreed changes after the interdisciplinary consolidation in the outpatient setting.

The assessment detected a high proportion (80.6%) of patients with at least one change between the physician-documented MP and the CMP. In total, one-third of all drugs were changed. Accordingly, the cardiovascular medication was changed in a comparable proportion (30.3%). Analyzing the impact of the interdisciplinary consolidation on the patient’s current drug regimen, 74.2% of patients had at least one change, affecting about one-quarter of their drugs.

Our approach varies from other studies analyzing the variations in the drug regimens. The studies so far usually report on the discrepancies between the physician-documented and patient-stated medications, for example, in the outpatient setting (Bedell et al., 2000; Bikowski et al., 2001; Waltering et al., 2015; Rose et al., 2018). From these findings, the authors derive or measure the clinical risks these discrepancies might lead to (Waltering et al., 2015; Rose et al., 2018). We extended these important findings by comparing the physician-documented MP with the interdisciplinary CMP. In addition, we compared the patient-stated medication with the CMP. The identified deviations between the MP or the patient-stated medication and the CMP were interpreted as agreed changes based on the decisions of the healthcare professionals and in agreement with the patient. Thus, we focused on the interdisciplinary care after the discrepancies had been identified in a medication review, aiming to improve patient safety and medication effectiveness.

In PHARM-CHF, this was especially important as the patients in the pharmacy care group received their medication in weekly dosing aids according to the interdisciplinary CMP. By relying solely on the initial physician-documented MP, there would have been unintended additions, omissions, and modifications in the drug regimen of CHF patients.

As the results in this CHF cohort show, diuretics, beta-blockers, and agents acting on the renin-angiotensin-aldosterone system (RAAS) were largely affected by the changes between the MP and the CMP. Thus, physicians and pharmacists frequently saw the need for the modification of the drug regimen, especially in this HF-related medication.

### Interdisciplinary Approach Leads to Changes in the Drug Regimen

Other studies analyzing the discrepancies between the drugs documented by the physicians and the actually used medication stated by the patients, found substantial disagreements between both lists (Bedell et al., 2000; Bikowski et al., 2001; Schnipper et al., 2018). However, the strategies for improving the accuracy in the outpatient setting were not investigated.

Van Stiphout et al. conducted a non-interdisciplinary approach where the physicians received intensive education and an e-learning training (van Stiphout et al., 2018). However, this training had no effect on decreasing the number of discrepancies (van Stiphout et al., 2018).

The interdisciplinary pharmacist-physician collaboration is able to improve the accuracy of medication lists (Arnold et al., 2015; Holland, 2015). Numerous studies have shown that the pharmacist recommendations for the changes based on a medication review had a positive impact on the patient’s pharmacotherapy. These studies also determined high acceptance rates of 64% to over 90% by the physicians for the changes proposed by the pharmacists (Krska et al., 2001; Kiel and Phillips, 2018).

We tested community pharmacists performing a medication review and subsequently collaborating with the physician to develop an interdisciplinary CMP. The medication review entails patient interviews, which is crucial, as this has been shown to be a successful strategy to optimize and complete the information about the current drug regimen (Tulner et al., 2009).

The drug information stated by the patient had a considerable impact on the CMP, as approximately 68% of the changes were based on the patient’s reporting during the medication review. Therefore, this interdisciplinary approach also involving patients, harmonized and optimized the drug regimen as patient safety relies on a correct medication plan.

### Frequencies of Medication Changes and Discrepancies in Literature

We detected 80.6% of patients with at least one medication change between the physician-documented MP and the CMP. A comparison of these findings with previous studies is difficult, as most of the trials analyzed the discrepancies between the MP and the patient-stated medication or the electronic pharmacy records. Discrepancies between patient-stated medication and physician-documented MP were observed in 76% to 96% of patients (Bedell et al., 2000; Waltering et al., 2015; Rose et al., 2018; van Stiphout et al., 2018). In contrast to the reported discrepancies, which may be unintended due to missing communication or lack of knowledge by the physicians, the changes between the MP/patient-stated medication and the CMP in our study are an agreed result of the interdisciplinary consolidation.

### Predictors for Medication Changes

We determined no association between the patient’s age, sex, care level, NYHA class, and the number of medication changes. We observed, however, that the number of drugs as well as the specialty of the physician affected the number of changes. For the discrepancies, Bedell and others reported that the patient age and the number of drugs were significant predictors (Bedell et al., 2000). However, we did not find age-specific differences in the number of changes. This could be due to a high mean and a narrow distribution of age in our cohort and the general drug burden of CHF patients.

Comparable to our findings, other studies reported that age and sex were predominantly no predictors for the discrepancies (Hias et al., 2017; Giannini et al., 2019). In addition, other studies showed that more discrepancies happened when the documenting physician was a specialist (Bedell et al., 2000; Waltering et al., 2015). This could explain why more changes occurred in the CMP if a cardiologist documented medications, as these MP were less accurate and thus had to be revised more extensively.

In addition, we observed fewer changes for the patients taking part in an HF-DMP. The primary goal of this DMP for the patients with HF is to prevent hospitalizations and improve the patient’s quality of life (Poelzl et al., 2020). Common to all DMP is comprehensive patient education, monitoring of symptoms, and optimization of treatment based on established guidelines (Moertl et al., 2017; Poelzl et al., 2020). The latter could explain the lower number of changes observed for the patients participating in an HF-DMP. According to the European Society of Cardiology, the DMPs for the patients with HF are strongly recommended (recommendation class 1, level of evidence A) (Ponikowski et al., 2016). Thus, we assume that particularly the patients with a high number of drugs medically attended by specialists and not included in an HF-DMP may benefit the most from this physician-pharmacist collaboration.

### Cardiovascular Medication

Patients with CHF are prone to discrepancies in the different documenting of their drug regimen (Ekedahl et al., 2011). This may be due to the variety of caregivers involved in treating these patients and the high number of drugs used to treat the HF signs and symptoms, as well as the frequent comorbidities. The main reason for hospitalizations is the HF decompensation, often resulting from medication non-adherence (Kobayashi et al., 2020). One of the reasons for non-adherence in CHF patients is, among others, the complexity of the drug regimen (Forsyth et al., 2019). Thus, it is important to harmonize the physician-documented and patient-stated drug regimens to support CHF patients in their pharmacotherapy.

Common deviations in cardiovascular medication are omissions and differing doses of antihypertensives, for example, RAAS inhibitors and beta-blockers (Waltering et al., 2015; Rose et al., 2018). These deviations held the risk of undetected causes for hospitalizations, medication errors, inappropriate prescribing, and other DRP (Rose et al., 2018; Goyal et al., 2020).

A pharmacist-led medication review as in our study is able to identify these problems and leads to discussions with the physicians about the necessary adjustments (Waltering et al., 2015; Rose et al., 2018; Sell and Schaefer, 2020). This process led to changes in 30.3% of the HF medication between the MP and the CMP. In 71.7%, the changes were based on the information stated by the patient. Additionally, the patient-stated drug regimens were adjusted to minimize the risk of DRP. This shows that various changes of the physician-documented MP and patient-stated medication are needed to optimize and harmonize the HF drug regimen, potentially increasing the patient safety.

### Strengths and Limitations

#### Strengths

This study adds to the existing analyses on the discrepancies between the physician-documented and patient-stated medications by further investigating the changes in medication which result from identifying these discrepancies. Thus, it enables an insight into the collaboration of physicians and pharmacists optimizing and harmonizing the medication of HF patients, which is scarce in the available literature. Another strength is the consideration of a vulnerable and multimorbid group of patients relying on polypharmacy for treatment and it is especially prone to drug discrepancies. This required the analysis of complex drug regimens and added to the challenge of interdisciplinary cooperation, which was successfully managed in an outpatient setting. Finally, due to thorough documentation, the data revealed the enormous impact of the patient’s role in the changes made by the healthcare professionals.

#### Limitations

We analyzed the changes between physician-documented MP and patient-stated drug regimen and interdisciplinary CMP based on the medication documentation in PHARM-CHF. Statements the interdisciplinary team explaining the performed changes were not collected by default. Thus, we were often limited in interpreting the reasons for the necessary adjustments. However, because most of the changes were omissions, additions, and modifications according to the patient-stated medication in the review, the explanation of harmonization and optimization is plausible.

Different pharmacists performed the medication review and the consolidation was performed in various interdisciplinary teams. Therefore, a personal bias in the estimation of necessary adjustments cannot be excluded. To standardize these processes, we provided an online tool for the communication of medication lists and of the identified discrepancies and DRP between the professions. Additionally, the study pharmacists were trained consistently by the investigators in the performance of the medication review.

The sample size and power computations of the study were performed on the primary efficacy outcome of PHARM-CHF that was not the number of changes but adherence to HF medications (Schulz et al., 2019). Therefore, our statistics on the predictors of changes have to be interpreted with care. However, our results correlate with the findings in the current literature. In addition, we could not check for an association between the patient’s level of education or the patient’s income and the number of changes, because these data were not collected in PHARM-CHF. We also did not analyze whether the observed changes affected the primary endpoint of the PHARM-CHF randomized controlled trial. This study only examined the process of a one-time medication review followed by an interdisciplinary consolidation of the MP. The subsequent interdisciplinary adjustments in the medication were not analyzed and should be considered in future studies.

A narrow age distribution was caused by the inclusion criteria of the PHARM-CHF study in which patients had to be 60 years and older. However, patients with HF are generally older so the study group is an accurate sample.

## Conclusion

Interdisciplinary consolidation of MP led to frequent and potentially clinically relevant changes in the medication for elderly CHF patients. Nearly one-third of drugs in the initially physician-documented MP varied from the CMP. The need for changes increased with the number of medications identifying a subgroup with high risk which can benefit from specific support. In addition, the initial MP from cardiologists required more adjustments compared to those documented by GPs. Patients not participating in an HF-DMP were mainly affected by medication adjustments. A pharmacist-led medication review allowed the involvement of patient’s current pharmacotherapy in the process of developing a coherent CMP. This harmonized and optimized the drug regimen. Both sources, the physician-documented medication lists and the current drug regimen stated by the patient, should be considered for an optimal CMP. To assure the effectiveness of the medication and patient safety, a regularly updated CMP should be easily accessible to all individuals involved in the pharmacotherapy, ideally in an electronic format.

## Data Availability

The raw data supporting the conclusions of this article will be made available by the authors, at reasonable request.

## Abbreviations

CHF, chronic heart failure; CMP, interdisciplinary consolidated medication plan; DRP, drug-related problem; GP, general practitioner; HF, heart failure; HF-DMP, heart failure disease management program; IQR, interquartile range; MP, physician-documented medication plan; MRA, mineralocorticoid receptor antagonists; RAAS, agents acting on the renin-angiotensin-aldosterone system.

## Appendix

PHARM-CHF investigators: Stefan D. Anker (Berlin), Michael Böhm (Homburg/Saar), Friedrich Koehler (Berlin), Dietmar Trenk (Freiburg/Bad Krozingen), Lea Botermann, Katrin Krueger, Nicole Krügerke, Judith Mantzke, Natalie Parrau, Dorothea Strauch (Berlin), Angelika Wachter (Homburg/Saar), Kati Fikenzer, Rolf Wachter (Leipzig), Peter Ihle, Ingrid Schubert (Cologne), and Charlotte Kloft (Berlin).

## References

[B1] Almanasreh E. Moles R. Chen T. F. (2019). The Medication Discrepancy Taxonomy (MedTax): The Development and Validation of a Classification System for Medication Discrepancies Identified through Medication Reconciliation. Res. Soc. Adm Pharm 16, 142–148. 10.1016/j.sapharm.2019.04.005 31015008

[B2] Arnold M. E. Buys L. Fullas F. (2015). Impact of Pharmacist Intervention in Conjunction with Outpatient Physician Follow-Up Visits after Hospital Discharge on Readmission Rate. Am. J. Health Syst. Pharm. 72, 36–42. 10.2146/sp150011 25991594

[B3] Bedell S. E. Jabbour S. Goldberg R. Glaser H. Gobble S. Young-Xu Y. (2000). Discrepancies in the Use of Medications: Their Extent and Predictors in an Outpatient Practice. Arch. Intern. Med. 160, 2129–2134. 10.1001/archinte.160.14.2129 10904455

[B4] Bikowski R. M. Ripsin C. M. Lorraine V. L. (2001). Physician-patient Congruence Regarding Medication Regimens. J. Am. Geriatr. Soc. 49, 1353–1357. 10.1046/j.1532-5415.2001.49265.x 11890495

[B5] Ekedahl A. Brosius H. Jönsson J. Karlsson H. Yngvesson M. (2011). Discrepancies between the Electronic Medical Record, the Prescriptions in the Swedish National Prescription Repository and the Current Medication Reported by Patients. Pharmacoepidemiol. Drug Saf. 20, 1177–1183. 10.1002/pds.2226 21858899

[B6] Forsyth P. Richardson J. Lowrie R. (2019). Patient-reported Barriers to Medication Adherence in Heart Failure in Scotland. Int. J. Pharm. Pract. 27, 443–450. 10.1111/ijpp.12511 30675955

[B7] Giannini O. Rizza N. Pironi M. Parlato S. Waldispühl Suter B. Borella P. (2019). Prevalence, Clinical Relevance and Predictive Factors of Medication Discrepancies Revealed by Medication Reconciliation at Hospital Admission: Prospective Study in a Swiss Internal Medicine ward. BMJ open 9, e026259. 10.1136/bmjopen-2018-026259 PMC6538074 31133583

[B8] Goyal P. Kneifati-Hayek J. Archambault A. Mehta K. Levitan E. B. Chen L. (2020). Prescribing Patterns of Heart Failure-Exacerbating Medications Following a Heart Failure Hospitalization. JACC Heart Fail. 8, 25–34. 10.1016/j.jchf.2019.08.007 31706836 PMC7521627

[B9] Griese-Mammen N. Hersberger K. E. Messerli M. Leikola S. Horvat N. van Mil J. W. F. (2018). PCNE Definition of Medication Review: Reaching Agreement. Int. J. Clin. Pharm. 40, 1199–1208. 10.1007/s11096-018-0696-7 30073611

[B10] Groenewegen A. Rutten F. H. Mosterd A. Hoes A. W. (2020). Epidemiology of Heart Failure. Eur. J. Heart Fail. 22, 1342–1356. 10.1002/ejhf.1858 32483830 PMC7540043

[B11] Hias J. van der Linden L. Spriet I. Vanbrabant P. Willems L. Tournoy J. (2017). Predictors for Unintentional Medication Reconciliation Discrepancies in Preadmission Medication: a Systematic Review. Eur. J. Clin. Pharmacol. 73, 1355–1377. 10.1007/s00228-017-2308-1 28744584

[B12] Holland D. M. (2015). Interdisciplinary Collaboration in the Provision of a Pharmacist-Led Discharge Medication Reconciliation Service at an Irish Teaching Hospital. Int. J. Clin. Pharm. 37, 310–319. 10.1007/s11096-014-0059-y 25595443

[B13] Imfeld-Isenegger T. L. Pham M. B. T. Stämpfli D. Albert V. Almanasreh E. Moles R. (2020). Medication Discrepancies in Community Pharmacies in Switzerland: Identification, Classification, and Their Potential Clinical and Economic Impact. Pharmacy (Basel) 8, 36. 10.3390/pharmacy8010036 PMC7151719 32182863

[B14] Kiel W. J. Phillips S. W. (2018). Impact of Pharmacist-Conducted Comprehensive Medication Reviews for Older Adult Patients to Reduce Medication Related Problems. Pharmacy (Basel) 6, 2. 10.3390/pharmacy6010002 PMC5874541 29301226

[B15] Kobayashi M. Voors A. A. Girerd N. Billotte M. Anker S. D. Cleland J. G. (2020). Heart Failure Etiologies and Clinical Factors Precipitating for Worsening Heart Failure: Findings from BIOSTAT-CHF. Eur. J. Intern. Med. 71, 62–69. 10.1016/j.ejim.2019.10.017 31708361

[B16] Krska J. Cromarty J. A. Arris F. Jamieson D. Hansford D. Duffus P. R. (2001). Pharmacist-led Medication Review in Patients over 65: a Randomized, Controlled Trial in Primary Care. Age Ageing 30, 205–211. 10.1093/ageing/30.3.205 11443021

[B17] Laufs U. Griese-Mammen N. Krueger K. Wachter A. Anker S. D. Koehler F. (2018). PHARMacy-Based Interdisciplinary Program for Patients with Chronic Heart Failure (PHARM-CHF): Rationale and Design of a Randomized Controlled Trial, and Results of the Pilot Study. Eur. J. Heart Fail. 20, 1350–1359. 10.1002/ejhf.1213 29846031

[B18] Moertl D. Altenberger J. Bauer N. Berent R. Berger R. Boehmer A. (2017). Disease Management Programs in Chronic Heart Failure : Position Statement of the Heart Failure Working Group and the Working Group of the Cardiological Assistance and Care Personnel of the Austrian Society of Cardiology. Wien Klin Wochenschr 129, 869–878. 10.1007/s00508-017-1265-0 29080104 PMC5711993

[B19] Poelzl G. Fetz B. Altenberger J. Fritsch M. Auer J. Stachl E. (2020). Heart Failure Disease Management Programs in Austria 2019 : A Systematic Survey of the Heart Failure Working Group and the Working Group for Cardiological Assistance and Care Personnel of the Austrian Society of Cardiology. Wien Klin Wochenschr 132, 310–321. 10.1007/s00508-020-01615-y 32072313 PMC7297701

[B20] Ponikowski P. Voors A. A. Anker S. D. Bueno H. Cleland J. G. F. Coats A. J. S. (2016). 2016 ESC Guidelines for the Diagnosis and Treatment of Acute and Chronic Heart Failure: The Task Force for the Diagnosis and Treatment of Acute and Chronic Heart Failure of the European Society of Cardiology (ESC). Developed with the Special Contribution of the Heart Failure Association (HFA) of the ESC. Eur. Heart J. 37, 2129–2200. 10.1093/eurheartj/ehw128 27206819

[B21] Rose O. Jaehde U. Köberlein-Neu J. (2018). Discrepancies between home Medication and Patient Documentation in Primary Care. Res. Soc. Adm Pharm 14, 340–346. 10.1016/j.sapharm.2017.04.003 28412152

[B22] Schmiemann G. Bahr M. Gurjanov A. Hummers-Pradier E. (2012). Differences between Patient Medication Records Held by General Practitioners and the Drugs Actually Consumed by the Patients. Int. J. Clin. Pharmacol. Ther. 50, 614–617. 10.5414/CP201682 22762854

[B23] Schnipper J. L. Mixon A. Stein J. Wetterneck T. B. Kaboli P. J. Mueller S. (2018). Effects of a Multifaceted Medication Reconciliation Quality Improvement Intervention on Patient Safety: Final Results of the MARQUIS Study. BMJ Qual. Saf. 27, 954–964. 10.1136/bmjqs-2018-008233 30126891

[B24] Schulz M. Griese-Mammen N. Anker S. D. Koehler F. Ihle P. Ruckes C. (2019). Pharmacy-based Interdisciplinary Intervention for Patients with Chronic Heart Failure: Results of the PHARM-CHF Randomized Controlled Trial. Eur. J. Heart Fail. 21, 1012–1021. 10.1002/ejhf.1503 31129917

[B25] Schulz M. Griese‐Mammen N. Schumacher P. M. Anker S. D. Koehler F. Ruckes C. (2020). The Impact of Pharmacist/physician Care on Quality of Life in Elderly Heart Failure Patients: Results of the PHARM‐CHF Randomized Controlled Trial. ESC Heart Fail. 7, 3310–3319. 10.1002/ehf2.12904 PMC7754956 32700409

[B26] Sell R. Schaefer M. (2020). Prevalence and Risk Factors of Drug-Related Problems Identified in Pharmacy-Based Medication Reviews. Int. J. Clin. Pharm. 42, 588–597. 10.1007/s11096-020-00976-8 32026355 PMC8452550

[B27] Tsuyuki R. T. McKelvie R. S. Arnold J. M. Avezum A A. Barretto A. C. Carvalho A. C. (2001). Acute Precipitants of Congestive Heart Failure Exacerbations. Arch. Intern. Med. 161, 2337–2342. 10.1001/archinte.161.19.2337 11606149

[B28] Tulner L. R. Kuper I. M. Frankfort S. V. van Campen J. P. Koks C. H. Brandjes D. P. (2009). Discrepancies in Reported Drug Use in Geriatric Outpatients: Relevance to Adverse Events and Drug-Drug Interactions. Am. J. Geriatr. Pharmacother. 7, 93–104. 10.1016/j.amjopharm.2009.04.006 19447362

[B29] van Stiphout F. Zwart-van Rijkom J. E. F. Versmissen J. Koffijberg H. Aarts J. E. C. M. van der Sijs I. H. (2018). Effects of Training Physicians in Electronic Prescribing in the Outpatient Setting on Clinical, Learning and Behavioural Outcomes: a Cluster Randomized Trial. Br. J. Clin. Pharmacol. 84, 1187–1197. 10.1111/bcp.13540 29399852 PMC5980599

[B30] Waltering I. Schwalbe O. Hempel G. (2015). Discrepancies on Medication Plans Detected in German Community Pharmacies. J. Eval. Clin. Pract. 21, 886–892. 10.1111/jep.12395 26139566

[B31] Yates L. Valente M. Wadsworth C. (2020). Evaluation of Pharmacist Medication Review Service in an Outpatient Heart Failure Clinic. J. Pharm. Pract. 33, 820–826. 10.1177/0897190019842696 31057060

