# Supplementary material for: Interdisciplinary Physician-Pharmacist Medication Review for Outpatients With Heart Failure: A Subanalysis of the PHARM-CHF Randomized Controlled Trial
Source: Front Pharmacol. 2021 Sep 7;12:712490. doi: 10.3389/fphar.2021.712490 (PMC8453145; doi:10.3389/fphar.2021.712490)
Supplement: Supplementary file 1 [file DataSheet1.PDF]

Checklist for Drug-related Problems (DRP)

1. **Are drugs missing in the patient-stated medication that are listed on the physician-documented medication plan (MP)?**
   - ☐ No
   - ☐ Yes – please record on documentation sheet for DRP

2. **Are drugs named in the patient-stated medication (Rx or non-prescribed) that are not listed on the MP?**
   - ☐ No
   - ☐ Yes – please record on documentation sheet for DRP

3. **Are there differences in drug dosing between MP and patient-stated medication?**
   - ☐ No
   - ☐ Yes – please record on documentation sheet for DRP

4. **Are there unintended duplicates (two or more drugs with the same active ingredient or from the same drug class)?**
   - ☐ No
   - ☐ Yes – please record on documentation sheet for DRP

5. **Are there potentially severe drug/drug-interactions** (classified as: serious consequences probable – contraindicated; under certain conditions monitoring or adjustment required) **that resulted in an intervention by you or had to be discussed with the physician?**
   - ☐ No
   - ☐ Yes – please record on documentation sheet for DRP

6. **Does the patient use drugs that are contraindicated according to the national practice guideline for chronic heart failure (NVL)?**
   - ☐ No
   - ☐ Yes – please record on documentation sheet for DRP

7. **Did you identify possible adverse drug effects?**
   - ☐ No
   - ☐ Yes – please record on documentation sheet for DRP

8. **Did you identify medication non-adherence (drugs not taken – intentionally or unintentionally)?**
   - ☐ No
   - ☐ Yes – please record on documentation sheet for DRP

9. **Did you identify drug handling problems?**
   - ☐ No
   - ☐ Yes – please record on documentation sheet for DRP

10. **Does dosing require splitting of tablets that shall not be splitted?**
    - ☐ No
    - ☐ Yes – please record on documentation sheet for DRP

11. **Did you identify further DRP?**
    - ☐ No
    - ☐ Yes – please record on documentation sheet for DRP

© ABDA
